# First Complete Genome Sequence of a Hare Calicivirus Strain Isolated from *Lepus europaeus*

**DOI:** 10.1128/MRA.01224-18

**Published:** 2018-12-06

**Authors:** Clément Droillard, Evelyne Lemaitre, Marina Chatel, Jean-Sébastien Guitton, Stéphane Marchandeau, Nicolas Eterradossi, Ghislaine Le Gall-Reculé

**Affiliations:** a Avian and Rabbit Virology Immunology Parasitology Unit, Ploufragan-Plouzané-Niort Laboratory, French Agency for Food, Environmental and Occupational Health & Safety (Anses), Ploufragan, France; b Sedentary Small Fauna Unit, Research and Expertise Department, National Hunting and Wildlife Agency (ONCFS), Nantes, France; KU Leuven

## Abstract

The first full-genome sequence of a hare calicivirus (HaCV), recently characterized as a novel member of the *Caliciviridae*, is described. This presumed nonpathogenic lagovirus is 7,433 nucleotides long, shows the same genomic organization as that of other lagoviruses, and has the highest nucleotide identity (79%) with pathogenic European brown hare syndrome viruses.

## ANNOUNCEMENT

The *Caliciviridae* family comprises five assigned genera, Norovirus, Vesivirus, Sapovirus, Lagovirus, and Nebovirus (1). The Lagovirus genus contains a single species, named Lagovirus europaeus, which infects leporids. Its isolates are divided into two genogroups, with GI corresponding to rabbit hemorrhagic disease virus (RHDV)-related viruses, and GII. GII contains the pathogenic European brown hare syndrome virus (EBHSV, GII.1 genotype) and the hare calicivirus (HaCV), an unassigned genotype because still based on a single available capsid protein (VP60) sequence (2). HaCV, first detected in 2014 from healthy hares (Lepus europaeus) born and reared in an Italian farm, is presumed to be nonpathogenic (3).

In 2015 in France, HaCV strain E15-431 was detected by reverse transcription-PCR (RT-PCR) using PCR primers located in conserved regions for lagoviruses within the VP60 gene (4), in a hunted wild hare collected as part of the ECALEP European project. This project aimed at understanding the origin of pathogenic lagoviruses (5). Viral RNA was extracted from a piece of duodenum (tissue and content) and was reverse transcribed to cDNA using oligo(dT) and Maxima reverse transcriptase (Thermo Scientific). The genome was PCR amplified with the Expand high-fidelity PCR system (Roche) using combinations of primers designed in conserved regions of the lagovirus genomes or by using a genome-walking strategy. Eight overlapping fragments between 800 and 3,000 bp in length were generated. They covered the entire coding sequence. PCR products were sequenced with an Applied Biosystems 3130 Sanger-based genetic analyzer in both directions, and the consensus sequence was compiled using Vector NTI Advance 11. To confirm the obtained sequence, the amplification process was repeated but this time using primer pairs designed on the consensus. Five overlapping fragments were generated of approximately 2,000 bp, and these were again sequenced in both directions using Sanger technology. The genome extremities were acquired using the rapid amplification of cDNA ends (RACE) method (6). The complete genome sequence was compiled using Vector NTI Advance 11. Pairwise distance analysis was performed using the MEGA7 software (7).

The E15-431 genome sequence is 7,433 nucleotides (nt) in length, excluding the poly(A) tail. BLAST analysis of the entire genome shows that the most closely related viruses are the GII.1 lagoviruses, with 79% identity with the four characterized GII.1 entire genomes (GenBank accession numbers KC832838, KC832839, Z69620, and MF356366). The nearest GI lagovirus shows 73% identity (GenBank accession number KX357675). The genomic RNA is organized into two open reading frames (ORFs). ORF1 is 7,008 nt long and encodes a 2,335-amino-acid (aa)-long protein, and ORF2 is 345 nt long and encodes a 114-aa-long protein. The length of the 5′ untranslated region (UTR) is 9 nt, and that of the 3′ UTR is 79 nt, excluding the poly(A) tail. The deduced ORF1 protein sequence was compared with a GI.1 polyprotein sequence for which cleavage sites have been experimentally described (8). From this comparison, it was clear that the deduced ORF1 of HaCV had the same order of nonstructural and structural proteins obtained after the proteolytic processing of the polyprotein. The nonstructural proteins p16, p23, helicase, p29, VPg, 3C-like protease, and RNA-dependent RNA polymerase (RdRp) are 139, 224, 349, 275, 115, 143, and 516 aa long, respectively. The major structural protein VP60 is 574 aa long. However, one out of the seven cleavage sites is different, since the E^1251^-T^1252^ site is replaced by E^1245^-N^1246^, as reported for some GII.1 sequences (9, 10). In addition, the comparison of the E15-431 polyprotein with the four GII.1 ones shows a single amino acid insertion in HaCV p16. ORF2 encodes a protein similar to the GII.1 minor structural protein VP10. Pairwise distance analysis with the available HaCV VP60 sequence (GenBank accession number KR230102) shows 84.7% nucleotide identity, underlying the high genetic distance between the two HaCV sequences. This study adds a new genomic sequence to the few sequences available on GII lagoviruses and will facilitate research on the phylogenetic relationships of lagoviruses and on the origin of their pathogenicity.

### Data availability.

The full-genome sequence of HaCV E15-431 has been deposited in GenBank under the accession number MH204883.

## References

[1] Clarke IN, Estes MK, Green KY, Hansman GS, Knowles NJ, Koopmans MK, Matson DO, Meyers G, Neill JD, Radford A, Smith AW, Studdert MJ, Thiel H-J, Vinjé J 2012 Virus taxonomy classification and nomenclature of viruses. *In* King AMQ, Adams MJ, Carstens EB, Lefkowitz EJ (ed), Ninth report of the International Committee on Taxonomy of Viruses. Elsevier, Academic Press, London, United Kingdom.

[2] Le Pendu J, Abrantes J, Bertagnoli S, Guitton JS, Le Gall-Reculé G, Lopes AM, Marchandeau S, Alda F, Almeida T, Celio AP, Barcena J, Burmakina G, Blanco E, Calvete C, Cavadini P, Cooke B, Dalton K, Delibes Mateos M, Deptula W, Eden JS, Wang F, Ferreira CC, Ferreira P, Foronda P, Goncalves D, Gavier-Widén D, Hall R, Hukowska-Szematowicz B, Kerr P, Kovaliski J, Lavazza A, Mahar J, Malogolovkin A, Marques RM, Marques S, Martin-Alonso A, Monterroso P, Moreno S, Mutze G, Neimanis A, Niedzwiedzka-Rystwej P, Peacock D, Parra F, Rocchi M, Rouco C, Ruvoen-Clouet N, Silva E, Silverio D, Strive T, Thompson G, 2017 Proposal for a unified classification system and nomenclature of lagoviruses. J Gen Virol 98:1658–1666. doi:10.1099/jgv.0.000840. 28714849

[3] Cavadini P, Molinari S, Pezzoni G, Chiari M, Brocchi E, Lavazza A, Capucci L 2015 Identification of a new non-pathogenic lagovirus in *Lepus europeaus*, p 76–77. *In* Albina E (ed), Changing viruses in a changing world. Xth International Congress for Veterinary Virology, Montpellier, France.

[4] Le Gall-Reculé G, Zwingelstein F, Fages MP, Bertagnoli S, Gelfi J, Aubineau J, Roobrouck A, Botti G, Lavazza A, Marchandeau S 2011 Characterisation of a non-pathogenic and non-protective infectious rabbit lagovirus related to RHDV. Virology 410:395–402. doi:10.1016/j.virol.2010.12.001. 21195443

[5] Esteves PJ, Abrantes J, Bertagnoli S, Cavadini P, Gavier-Widén D, Guitton JS, Lavazza A, Lemaitre E, Letty J, Lopes AM, Neimanis AS, Ruvoën-Clouet N, Le Pendu J, Marchandeau S, Le Gall-Reculé G 2015 Emergence of pathogenicity in lagoviruses: evolution from pre-existing nonpathogenic strains or through a species jump? PLoS Pathog 11:e1005087. doi:10.1371/journal.ppat.1005087. 26540662 PMC4634945

[6] Lemaitre E, Zwingelstein F, Marchandeau S, Le Gall-Reculé G 2018 First complete genome sequence of a European non-pathogenic rabbit calicivirus (lagovirus GI.3). Arch Virol 163:2921–2924. doi:10.1007/s00705-018-3901-z. 29978262 PMC6132933

[7] Kumar S, Stecher G, Tamura K 2016 MEGA7: Molecular Evolutionary Genetics Analysis version 7.0 for bigger datasets. Mol Biol Evol 33:1870–1874. doi:10.1093/molbev/msw054. 27004904 PMC8210823

[8] Meyers G, Wirblich C, Thiel HJ, Thumfart JO 2000 Rabbit hemorrhagic disease virus: genome organization and polyprotein processing of a calicivirus studied after transient expression of cDNA constructs. Virology 276:349–363. doi:10.1006/viro.2000.0545. 11040126

[9] Le Gall G, Huguet S, Vende P, Vautherot JF, Rasschaert D 1996 European brown hare syndrome virus: molecular cloning and sequencing of the genome. J Gen Virol 77:1693–1697. doi:10.1099/0022-1317-77-8-1693. 8760416

[10] Lopes AM, Gavier-Widén D, Le Gall-Reculé G, Esteves PJ, Abrantes J 2013 Complete coding sequences of European brown hare syndrome virus (EBHSV) strains isolated in 1982 in Sweden. Arch Virol 158:2193–2196. doi:10.1007/s00705-013-1714-7. 23640583

